# Nuclear Import of HIV-1

**DOI:** 10.3390/v13112242

**Published:** 2021-11-08

**Authors:** Qi Shen, Chunxiang Wu, Christian Freniere, Therese N. Tripler, Yong Xiong

**Affiliations:** Department of Molecular Biophysics and Biochemistry, Yale University, New Haven, CT 06511, USA; qi.shen@yale.edu (Q.S.); chunxiang.wu@yale.edu (C.W.); christian.freniere@yale.edu (C.F.); therese.tripler@yale.edu (T.N.T.)

**Keywords:** HIV-1 core, capsid, nuclear import, reverse transcription, uncoating, nuclear pore complex

## Abstract

The delivery of the HIV-1 genome into the nucleus is an indispensable step in retroviral infection of non-dividing cells, but the mechanism of HIV-1 nuclear import has been a longstanding debate due to controversial experimental evidence. It was commonly believed that the HIV-1 capsid would need to disassemble (uncoat) in the cytosol before nuclear import because the capsid is larger than the central channel of nuclear pore complexes (NPCs); however, increasing evidence demonstrates that intact, or nearly intact, HIV-1 capsid passes through the NPC to enter the nucleus. With the protection of the capsid, the HIV-1 core completes reverse transcription in the nucleus and is translocated to the integration site. Uncoating occurs while, or after, the viral genome is released near the integration site. These independent discoveries reveal a compelling new paradigm of this important step of the HIV-1 life cycle. In this review, we summarize the recent studies related to HIV-1 nuclear import, highlighting the spatial–temporal relationship between the nuclear entry of the virus core, reverse transcription, and capsid uncoating.

## 1. Introduction

**Figure 1:**
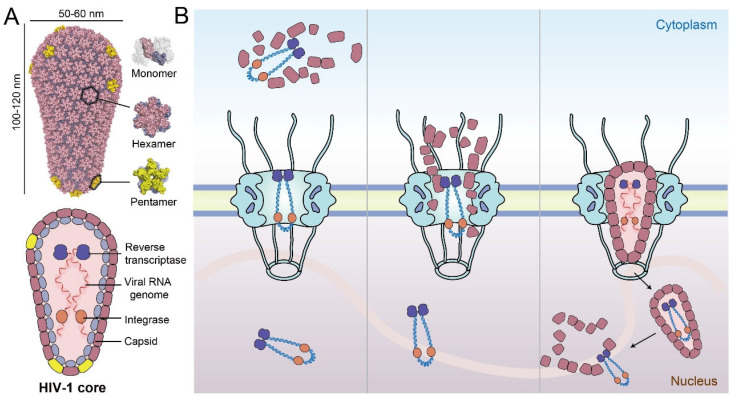
Schematic overview of the HIV-1 core and three potential models of nuclear import. (**A**) The cone-shaped viral capsid is assembled from CA hexamers and pentamers. The viral genome and necessary enzymes such as integrase and reverse transcriptase are housed inside the capsid. (**B**) Left panel—model of early uncoating in the cytosol after the virus core enters the cell; middle panel—capsid uncoating at the NPC; right panel—penetration of the intact HIV-1 core through the NPC and completion of reverse transcription and uncoating in the nucleus.

HIV-1 (AIDS) remains a worldwide threat to human health and society. As a retrovirus, the delivery of the viral genome to the nucleus is a necessary step in the infection process [1]. The HIV-1 genome and its essential enzymes for replication, such as reverse transcriptase and integrase, are housed inside the viral capsid (Figure 1A) to escape from degradation by innate immune sensors and cellular restriction factors [2,3,4]. HIV-1 capsid forms a fullerene cone structure, comprised of capsomeres of ~250 capsid protein (CA) hexamers and 12 CA pentamers [5,6,7,8,9]. HIV-1 gains access to the nucleus of non-dividing cells through the nuclear pore complex (NPC) [10]. However, the capsid cone extends ~120 nm in length and ~60 nm in width (Figure 1A) [7,9], which exceeds the generally accepted NPC central channel diameter of 40 nm [11]. This size difference, along with some supporting evidence, led to the commonly held belief that uncoating of the capsid is a necessary step in the cytosol, prior to nuclear import [4,12]. Due to conflicting experimental evidence, a longstanding debate exists concerning when and where capsid uncoating occurs [4,12,13].

To date, there are three major models of HIV-1 uncoating and nuclear import. The first proposes that early uncoating starts almost immediately after HIV-1 core enters the cytoplasm following virus–cell membrane fusion (Figure 1B, left) [4,14,15]. However, subsequent studies show that a stable capsid is necessary for reverse transcription and viral infection efficiency, thus, suggesting that early uncoating reduces the infection rate [16,17]. Later, several groups proposed a second model based on live-cell single-virus imaging studies [12,18,19], where capsid uncoating initiates when the virus core is docked on the NPC (Figure 1B, middle). Very recently, a third model was presented that the intact or nearly intact HIV-1 core penetrates through the NPC and uncoating occurs near sites of integration in the nucleus [13,20]. This model was supported by direct visualization using cryo-electron tomography (CryoET), suggesting that intact HIV-1 core crosses the NPC, followed by uncoating only when inside the nucleus [21,22]. However, the mechanisms by which the intact capsid interacts with the nucleoporins (NUPs) in the central channel of the NPC and crosses the nuclear pore remain to be elucidated (Figure 1B right) [21]. This review summarizes the latest progress of research on HIV-1 nuclear entry and highlights the spatial–temporal relationship of critical steps between nuclear import, capsid uncoating, and reverse transcription. Moreover, we describe the virus–nucleoporin interaction and other host factor interactions that facilitate the nuclear import of HIV-1 core, detailing the relationship between these factors and their properties that affect the delivery of the viral core to the nucleus.

## 2. Intact HIV-1 Core Is Capable of Entering into the Nucleus

A longstanding paradigm of the HIV-1 life cycle depicted uncoating of the retroviral capsid in the cytoplasm [4,14,15] or at the nuclear envelope (NE) during nuclear import [12,19], but not in the nucleus. In 2020, Burdick et al. developed methods to study HIV-1 uncoating by directly labeling CA with GFP, facilitating the tracking of viral cores in live infected cells [13]. The method overcomes the limit of previous indirect CA detection methods, which may not accurately reflect the status of the capsid, due to accessibility issues [14,23] or fast dissociation rate [12,18]. Imaging and quantification of the directly labeled capsids demonstrated that intact (or nearly intact) viral cores enter the nucleus (Figure 1B, right) and uncoat less than 1.5 h before integration near their genomic integration sites [13]. Subsequently, the result was substantiated that the HIV-1 capsid maintains its integrity during nuclear entry, and macromolecules in the viral core are not released until the core translocates to the integration site inside of the nucleus [20]. In parallel, Zila et al. used correlative light and electron microscopy with subtomogram averaging to visualize the structural status of HIV-1 cores in infected T cells, where snapshots of viral nuclear entry were captured [21]. Intact virus cores were directly observed passing through the NPC and persisting in the nucleus (Figure 1B, right), while empty and disrupted capsid fragments were also detected inside the nucleus. The authors arrived at a model that proposed that the viral core releases its genome when broken at one end, rather than being completely disassembled into individual subunits [21]. These studies used independent approaches to come to the same paradigm-shifting conclusion: intact viral cores can pass through the nuclear pore (Figure 1B, right) [13,20,21]. However, either cryoET or live-cell tracking experiments are limited by the number of particles measured and the selection of cell types. More studies are needed to confirm whether these results faithfully represent the process of HIV-1 nuclear import.

## 3. Timeline of the HIV-1 Nuclear Import, Reverse Transcription, and Uncoating

**Figure 2:**
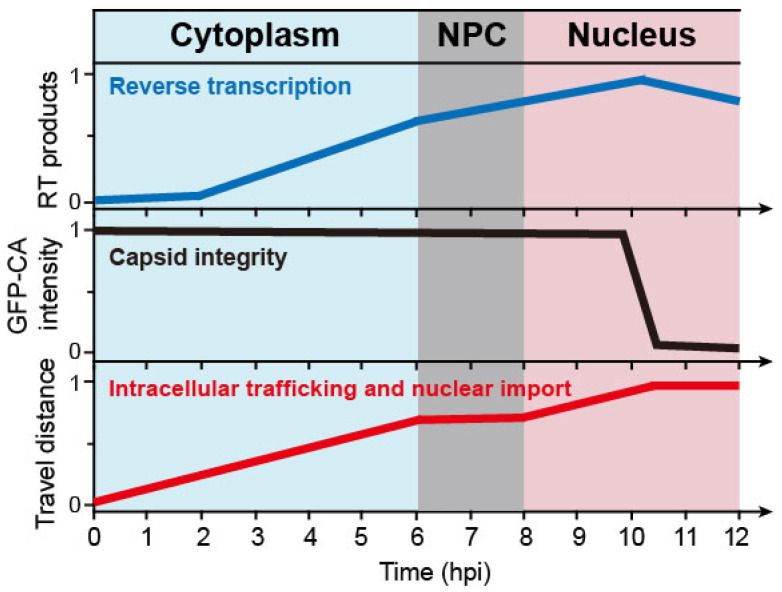
Schematic of the timelines for successful early-stage HIV-1 infection steps, including reverse transcription, uncoating, and nuclear import. The intact HIV-1 core penetrates the NPC independent of reverse transcription. The virus core completes reverse transcription and uncoats inside the nucleus. RT products—normalized amount of reverse transcription products; GFP-CA intensity—normalized GFP-CA signal intensity; travel distance—normalized distance from the periphery of the cytoplasm to the integration sites. The *x* and *y*-axis values are estimated from different sources [12,13,20,21,22,24,25,26,27,28,29,30].

The recent discovery that intact capsid can enter the nucleus through the NPC, was accompanied by other advances in understanding the related processes. Recent studies have presented a spatial–temporal relationship between the viral nuclear entry, reverse transcription (RT), and uncoating (Figure 2) [12,13,20,21,22,24,25,26,27,28,29,30]. The viral core moved from the periphery of the cytoplasm to the nucleus along microtubules, taking around 5–6 h to become docked to the cytoplasmic side of the NPC [13,26,27,28]. During this time, RT products were observed in the cytosol as early as 30 min after infection [12,29]. The early RT products dramatically increased after 5–6 h post infection [21]. Live-cell imaging results showed that the capsid stayed in the NPC for about 2 h before fully entering the nucleus, and retained its integrity during nuclear import [12]. About 8 h after infection, the virus core passed through the NPC [13,20]; meanwhile the late RT products continued to accumulate [12,21], suggesting that nuclear import does not depend on the completion of RT [29,30]. The latter point was also supported by experiments that showed that directly inhibiting reverse transcription does not affect the process of the virus core’s nuclear import [30]. Other recent studies also demonstrated that RT is finally completed in the nucleus [12,13,20,21]. At 2 h after nuclear import, and close to 10 h post incubation, the virus core reached a deeper region of the nucleus [13], localizing at transcriptionally active speckle-associated domains [3]. At this moment, late RT products were largely detected in the nucleus [21]. In vitro endogenous reverse transcription (ERT) assays also showed that the early RT products reached a peak at about 6 h, and the late products peaked at 8–10 h [24,25]. Following the completion of RT, it has been observed that one end of the virus capsid opened up and released the viral DNA [21,22]. Subsequently, the capsid may disassemble within 15 min, near the integration site (Figure 2) [13,20].

These data indicate that a successful HIV infection requires a virus core that: (1) travels to the NPC along microtubule networks with an intact capsid and conducts RT in the capsid; (2) docks to and passes through the NPC into the nucleus with an intact or largely intact capsid; (3) reaches nuclear speckles while completing RT; (4) rapidly uncoats near the integration site and releases viral DNA. Although compelling evidence has shaped a new landscape for our understanding of HIV-1 nuclear import, caveats should be noted due to technical limitations. Moreover, the timeline of the virus core that enters the nucleus may have a survivorship bias, emphasizing successful particles. For example, early uncoating of the capsid is frequently observed within the first hour after initiation of infection [4,14,15], while only intact capsid delivers its genome to the nucleus and completes integration for a successful infection [13,20,21]. Although it is justified to focus on the virus particles that lead to successful infection, some early or intermediate states of the “failed” particles may still be biologically relevant. In addition, it is still unknown whether HIV-1 has other ways to deliver its genome besides relying on the capsid. Many details about capsid uncoating, nuclear import, and RT are still uncertain and need further investigation.

## 4. The NPC Is Key to Viral Nuclear Import

### 4.1. The Diameter of the NPC’s Central Channel Can Dilate to ~64 nm

**Figure 3:**
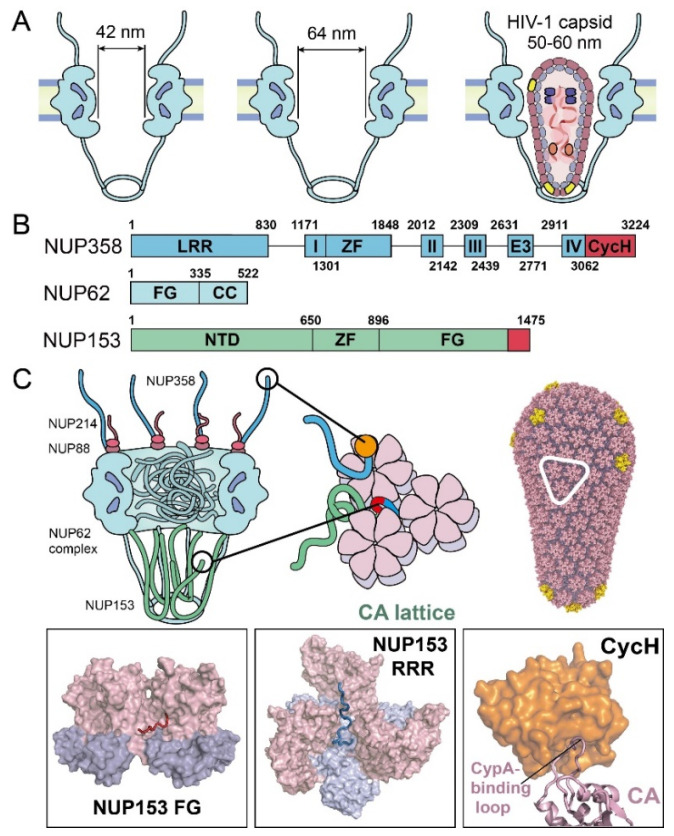
NPC components known to affect nuclear import of the HIV-1 core. (**A**) Schematics of the diameters of the NPC central channel and the size of HIV-1 capsid. (**B**) Domain organization of the major capsid-interacting NUPs, including NUP358, NUP153, and NUP62. Known CA-binding fragments are colored in red. Domain labels are as follows: LRR—leucine-rich region; roman numerals I–IV—Ran binding domains I–IV; ZF—zinc finger; E3—E3 ligase domain; CycH—cyclophilin homology domain; FG—phenylalanine-glycine repeats domain; CC—coiled-coil domain; NTD—N-terminal domain. (**C**) Summary of virus–nucleoporin interactions. The boxed insets show structural details of the interactions. The last FG-motif at NUP153 C-terminal region occupies the FG-binding pocket, formed at the NTD–CTD interface between adjacent CA monomers (PDB 4U0C). NUP153 “RRR” motif at its C-terminus binds at the CA tri-hexamer interface [57]. NUP358 CycH binds to the CypA-binding loop on CA (PDB 4LQW).

NPCs act as gatekeepers at the nuclear envelope, where they control the bidirectional exchange of molecules between the compartments of the nucleus and the cytoplasm [31,32]. They are the only known gateway for HIV-1 to deliver its viral genome into the nucleus and infect non-dividing cells [1,33]. The NPC is the largest macromolecular complex in the human cell (~110 MDa), which is composed of ~30 different nucleoporins (NUPs). Each NUP has 8–48 copies in the NPC that, together, build a basket-shaped structure [31,34,35,36] that houses a selective barrier in the central channel [37,38,39] (Figure 3A). The diameter of the human NPC central channel [11] was believed to be ~40 nm, which allows transport of cellular cargos [37,38,39], but was considered to be too narrow for the passage of an intact HIV-1 core. However, in analysis of cryo-electron tomograms of SupT1-R5 cells, Zila et al. showed that, regardless of the infection status, some NPCs have a significantly larger diameter of ~64 nm, which exceeds the width of the HIV-1 capsid (~55–60 nm) [21] (Figure 3A). It was suggested that the NPCs, purified from human cells in the form of nuclear envelope samples in previous structural analyses [11], may have lost inherent mechanical tension due to the sample preparation. These nuclear envelope-derived NPCs [11] likely represent a constricted state that may be more pertinent to stress release conditions [40]; whereas, the architecture of the NPC in HIV-1 infected and non-infected T cells, in situ, is more representative of the physiologic state for cargo transport. The NPC scaffold may rely on energy-dependent changes that could potentially dilate the channel [40]. The elasticity of the central channel of the NPC has been proposed to be indispensable to its biological functions [32,41,42,43,44]. Whether the ~64 nm width of the central channel is universal and reflects its natural state on other types of eukaryotic cells is still unknown; however, a recent report showed that intact HIV-1 capsid was detected in the nucleus of a wide range of cells beside SupT1-R5 [22].

### 4.2. Nucleoporins of the NPC Are Critical to HIV-1 Nuclear Import

To understand the nuclear entry of the capsid through the NPC, it is necessary to consider the abundance of nucleoporins (NUPs) with large, intrinsically disordered regions. NUPs, especially those lining the NPC central channel, are rich in repeated phenylalanine and glycine (FG)-motifs [32,34,35], which together act as a selective sieve to block macromolecules larger than 40 kD in molecular weight [37,38,39]. How the intact capsid crosses the crowded NPC central channel is still not clearly understood. NUP358, NUP214, NUP88, NUP62, and NUP153, located along the central channel, have been detected to interact with HIV-1 capsid [29,45,46,47,48] (Figure 3B and 3C). Among these NUPs, NUP358, NUP153—and, to a lesser extent—Nup62, have been shown to be the most important NUPs that are associated with HIV-1 nuclear import (Figure 3B and 3C).

NUP358 (also known as RanBP2) forms long filaments on the cytoplasmic side of the NPC [49] (Figure 3C). NUP358 has been demonstrated to directly interact with HIV-1 capsid and is likely involved in capturing the capsid to the cytoplasmic face of NPCs [50,51]. It is composed of 3224 residues that are rich in FG repeats and possess multiple domains, including a C-terminal cyclophilin homology domain (CycH) (Figure 3B) [52,53]. The CycH domain of NUP358 interacts with the HIV-1 capsid [50,51], similarly to Cyclophilin A (CypA, discussed more below), but with a much weaker affinity (dissociation constant Kd of ~200 μM) [54,55]. The CypA-binding loop of CA tucks into a hydrophobic pocket in the CycH domain (Figure 3C) [51]. This interaction is disrupted by CA mutations at positions G89 and P90 in the CypA-binding loop (Figure 3C) [45]. However, it has been shown that HIV-1 infection does not depend on the CycH of Nup358, suggesting that NUP358 may have other binding sites [56] to enhance the direct interaction with the capsid. Moreover, a previous study suggested that NUP358 relocates from the NPC to the cytosol post-infection to recruit the viral core for nuclear import in a kinesin KIF5B-dependent manner, via the microtubule [29]. However, it remains to be shown whether HIV highjacks NUP358 as an active delivery system to directly recruit the virus core onto the NPC.

NUP153 is a component of the NPC’s nuclear basket [58,59] (Figure 3C). It binds capsid directly and is essential for HIV-1 nuclear transport [47,60]. NUP153 contains 1475 residues, which includes 24 FG repeats in its C-terminal domain (CTD) (Figure 3B) [61,62]. Genetic analyses determined that the last C-terminal FG-motif of NUP153 is the main interactor, with the HIV-1 capsid [61]. Structural studies have shown that this NUP153 FG-motif is engaged at the NTD–CTD interface between two CA subunits of the hexamer, albeit with a relatively weak binding affinity (Kd) of ~50 μM [60,63]. It was recently identified that the C-terminal 65 residues of NUP153 interacted with CA assemblies beyond hexamers at a much higher affinity [57]. Notably, this strong interaction is achieved through a bipartite motif of NUP153, containing both the canonical FG-motif and an additional triple-arginine (RRR) motif [57]. Interestingly, the canonical FG-motif in NUP153 contributes modestly to the capsid interaction, and the other FG-repeats in NUP153 do not enhance binding. By contrast, the RRR-motif binds strongly at the interface of three CA hexamers, which is at the center of the three-fold symmetry axis on the assembled capsid. This binding mode suggests that, at least, a partially assembled capsid, beyond the CA hexamer, is required for stable NUP153–capsid interaction. Moreover, the presence of NUP153 appeared to stabilize the assembled CA lattice [57], which further suggests that NUP153 protects the integrity of the CA capsid during the capsid passage through the NPC. Given the location of NUP153 on the nuclear side of the NPC and the necessity of at least partially intact capsids for interaction, NUP153 may stabilize the capsid at the late stage of nuclear import.

NUP62 is an FG-NUP, localized at the NPC central transport channel (Figure 3C) [32,34]. It contains N-terminal FG-repeats and a C-terminal coiled-coil domain (Figure 3B) [64]. The coiled-coil region of NUP62 plays an important role in forming a trimeric complex of NUP62–NUP54–NUP58 [64,65], which constitutes the selective barrier of the NPC and thus plays a crucial role in the transport system [65]. Knockdown of NUP62-impaired viral integration, replication in HIV-1-susceptible cells [66], and NUP62 from cell lyate was co-pelleted with the capsid-mimicking CA nanotubes [48]. Recently, the kinetics of HIV-1 nuclear import were monitored with NPC blockade by an inducible homodimeric NUP62-fusion protein, and it was found that blocking the central channel prevented the virus core from passing through the NPC [29], even though a large number of NPC and capsid co-localized. Interestingly, NUP62 was observed to relocate from the NPC to cytoplasm post-infection, which suggests that HIV-1 infection may disrupt the central barrier of the NPC [29]. However, P90A and N74D mutants of the capsid are insensitive to a NUP62-mediated NPC blockade in cells, suggesting that HIV-1 may use other distinct nuclear import pathways during infection [29], which is consistent with the observations that similar mutations abolished (G89V) or reduced (N74D) the ability of the nuclear localized myxovirus resistance b (MxB) to inhibit the virus [67]. Moreover, there are also related studies suggesting that NUP62 directly interacts with integrase [66]. Further studies are needed to reveal more molecular-level mechanistic evidence to elucidate the NUP62 interaction with the viral core.

NUP214 and NUP88 form a stable subcomplex with NUP358 that helps form the cytoplasmic filaments of the NPC [68,69]. Both NUP214 and NUP88 are predicted to contain coiled-coil domains [70] that potentially interact with NUP62 [71]. NUP88 and NUP214 in cell lysates are co-pelleted with recombinant CA or CA–NC (nucleocapsid) nanotubes, pointing to their direct interaction with the capsid and their potential involvement in HIV-1 nuclear import [48,72]. However, a mechanistic understanding of the interactions and their roles in nuclear entry of the capsid are still lacking.

## 5. Other Host Factors Associated with HIV-1 Nuclear Import

Apart from the NUPs, many other cellular factors are identified to be important for the nuclear entry of the virus core. CypA has been proposed to stabilize capsid and protect the HIV-1 core from restriction factors [3,50]. Tripartite motif-containing protein 5 alpha (TRIM5α) and MxB [48,73,74,75] can directly target capsid as host antiviral restriction factors, thus blocking the virus core in the cytoplasm from the nuclear import process. Cleavage and polyadenylation specificity factor 6 (CPSF6) [63,76,77,78,79,80], some soluble nuclear transport receptors (NTRs) (including Karyopherin β2/Transportin-1 (TRN-1) [81] and Transportin-3 (TRN-SR2 or TNPO3)) [82,83], and SUN domain-containing proteins 1/2 (SUN1/2) [84,85] in the nuclear envelope are implicated to play roles in the HIV-1 passage through the NPC.

### 5.1. Cytoplasmic Protection and Restriction

**Figure 4:**
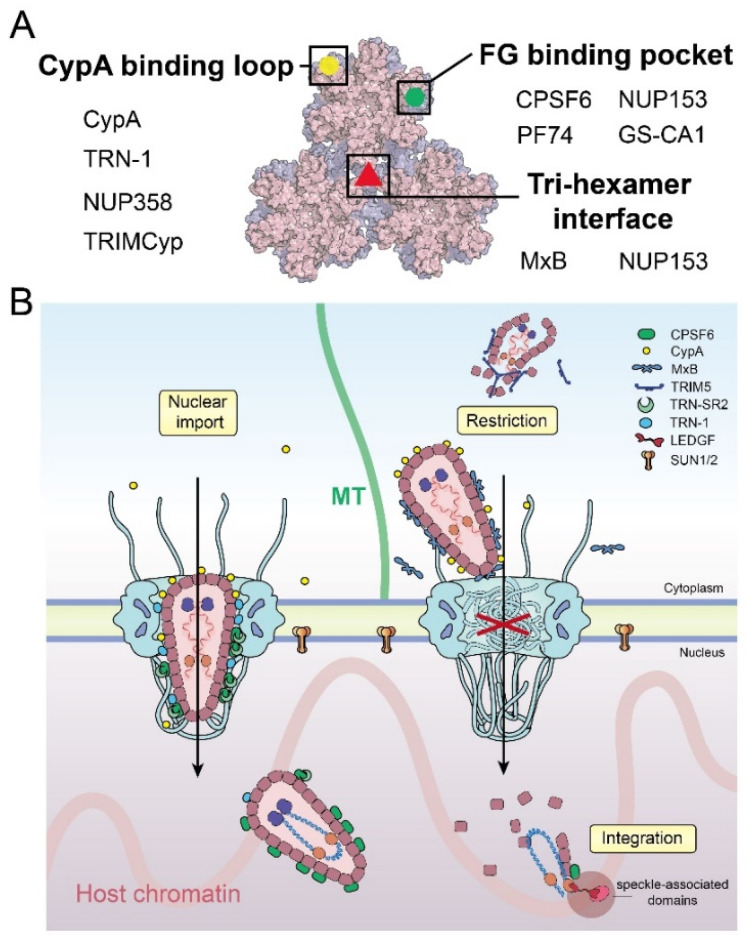
Factors affecting the nuclear import of HIV-1 capsid. (**A**) Schematic summary of the binding interfaces of HIV-1 capsid with host factors and inhibitors. Selected key interactors are listed. (**B**) Overview of HIV-1 capsid–host interactions during nuclear entry.

When HIV-1 releases the virus core into the cytoplasm, it needs to protect itself from restriction factors and innate immune defenses. CypA has been determined as a necessary protection factor and regulator for many early viral life cycle steps, from cellular trafficking to nuclear import [3,86]. It binds to the CypA-binding loop on CA and shelters and stabilizes the HIV-1 core to facilitate infection [87,88]. Recent structural work revealed additional binding sites over the assembled capsid’s CA di- and tri-hexamer interfaces in a curvature-dependent manner. [87,89]. These interactions indicate that CypA may serve as a CA lattice sensor to modulate the capsid stability and has the ability to block restriction factors, such as TRIM5α [30,90] and cyclic GMP-AMP (cGAS) [91]. Since CypA and NUP358 target the same CypA-binding loop of the CA protein (Figure 4A), the kinetics of the competitive binding between CypA and NUP358 to CA may affect the efficiency of the capsid docking on the NPC. The CA-binding affinity of the CycH domain of NUP358 alone is weaker than that of CypA [54,55]. It remains possible that binding from the 32 copies of NUP358 in the NPC enhances the affinity through an avidity effect, though this point warrants experimental clarification.

The ingress and nuclear transport of the virus core can be blocked by host restriction factors, such as TRIM5α [92] and MxB [75,93]. TRIM5α directly binds to the CA protein, and through self-oligomerization, forms a cage-like structure that covers the entire capsid [94] (Figure 4B). It was reported that TRIM5α causes early uncoating, which leads to the proteasomal degradation of the core and the inhibition of reverse transcription and nuclear import [92,95,96,97]. Other host factors, including tripartite motif-containing protein 34 (TRIM34) [98], tripartite motif-containing protein 11 (TRIM11) [99], and death domain-associated protein 6 (Daxx) [100], have also been shown to affect HIV-1 uncoating (reviewed in [101]). Near the NPC, MxB is recruited to the cytoplasmic face of the nuclear envelope, where a triple-arginine motif localized in the N-terminal of MxB can specifically bind to the CA tri-hexamer interface of the capsid and lead to the disruption of nuclear import (Figure 4) [75,102,103]. Interestingly, the triple-arginine motif was also found on the C-terminal of NUP153 [57]. As in MxB, this NUP153 motif specifically binds to the CA tri-hexamer interface but not to individual CA hexamers, which indicates that the assembled capsid lattice interface is necessary for nuclear translocation [104]. It was shown that MxB does not affect capsid docking to the NPC [30], but does affect capsid translocation to the nucleus. The observation that MxB and NUP153 share a common binding site on the CA lattice (Figure 4A) indicates that MxB may compete with NUP153 to block the viral core from entering the nucleus.

### 5.2. Nuclear Import

After the virus core successfully docks to the cytoplasmic side of the NPC, it needs the assistance of a variety of cellular factors to pass through the NPC central barrier made of various FG-NUPs, even though the size of the central channel does not appear to be an obstacle based on the latest studies [21,22].

TRN-1 is an NTR that was demonstrated to directly bind HIV-1 CA–NC nanotubes, recognizing the CypA-binding loop of CA as a nuclear localization signal (NLS) [81]. TRN-1 binding was proposed to facilitate capsid uncoating and transport to the nucleus. TRN-1 interacts with CA–NC nanotubes with a high affinity (K_d_ ~70 nM) but does not bind to individual CA–NC molecules [81]. This indicates that, like most of the other capsid-binding factors, an individual CA interaction is not sufficient and instead, there is likely a high-affinity binding site to the assembled capsid. Of note, the CypA-binding loop of CA is also the binding position of CypA and NUP358 (Figure 4A). It is intriguing to speculate that the interplay between these proteins could regulate the capsid entry into the nuclear pore.

CPSF6 plays an important role in modulating several steps in the HIV-1 life cycle, including the virus core cytoplasmic trafficking, nuclear import, and localization of the HIV-1 pre-integration complexes (PIC) inside the nucleus [63,76,77,78,79,80,86]. In the early stages of infection, CPSF6–capsid complexes have been observed to traffic on microtubules in the cytoplasm [86]. Excess cytoplasmic CPSF6 expression inhibits HIV-1 infection, consistent with earlier studies that a truncated cytoplasmic form of CPSF6 restricts HIV-1 [105]. CypA prevents capsid from prematurely binding CPSF6 and is consequently vital to the regulation of infection [86]. Higher-order CPSF6 binds and disrupts the assembled CA–NC nanotube in vitro [86,105]. Extensive studies show that the FG-motif of CPSF6 binds to the canonical FG-pocket on the CA hexamer at the NTD–CTD interface between two CA subunits with a weak affinity (K_d_ of ~80 μM) [63,77,79]. The CPSF6 FG-motif shares the same binding interface of CA hexamer with the NUP153 FG-motif (Figure 4A). Disruption of the CA–CPSF6 interaction results in capsid uncoating around the nuclear basket [21]. This suggests that the virus core could be released from the nuclear basket area by competitive binding between CPSF6 and NUP153. The interaction between CPSF6 and NUP153 may also lead to the remodeling of the capsid structure inside the NPC [57,106]. It has been reported that CPSF6 [105,107] and TRN-1 [81] deform or disrupt CA nanotubes in vitro. Consistently, Zila et al. found that most of the capsid retains a conical shape, but with a modified CA lattice, after passing through the NPC [21]. Furthermore, the integration of the viral DNA into the host genome is also dependent on the CPSF6–capsid interaction [76,77,78], in a yet-unknown manner.

The nuclear entry and translocation of the virus core to the transcriptionally active speckles in the nucleus may be facilitated by TRN-SR2 [107,108,109,110,111,112]. TRN-SR2 is a nuclear transport receptor of the serine or arginine-rich (SR) protein family [82,113], which also includes CPSF6 [108]. It was initially thought to interact with the HIV-1 integrase [114,115,116]; however, recent studies have suggested that TRN-SR2 facilitates the virus core nuclear import and localization of the pre-integration complex (PIC) by recognizing the SR2 region of CPSF6 [108]. Studies also showed that knockdowns of CPSF6 or TRN-SR2, or disruption of the recognition of CPSF6 by TRN-SR2, resulted in PIC and proviral accumulations in the peripheral region of the nucleus, rather than transcriptionally active speckles [12,13,21,77,80].

SUN1/2 proteins are anchored in the inner nuclear membrane and are essential components of the LINC (linker of nucleus and cytoskeleton) complex that mechanically tether the cytoskeleton to the nucleoskeleton [117]. The nucleoplasmic domains of SUN1 and SUN2 have been implicated in direct binding to HIV capsid [84,85]. Over expression experiments demonstrated that SUN1/2 restricts HIV-1 infectivity, while CRISPR-CAS9 studies revealed that knockout of SUN2 also inhibits HIV-1 infection [84,85,118,119]. These seemingly contradictory effects highlight the complex role of the finely balanced levels of SUN1/2 in facilitating nuclear transport of HIV-1 capsid. The mechanistic understanding of this process is largely lacking and must await future studies.

## 6. Summary and Perspective

Recent research advances have led to an emerging picture of the HIV-1 core entering the nucleus with an intact or largely intact capsid (Figure 4B), although many details still need to be elucidated. The capsid houses the viral genome and other necessary elements that infect host cells, such as the essential enzymes reverse transcriptase and integrase, together constituting the virus core that is delivered into the cell upon membrane fusion. In the cytoplasm, the virus core travels along microtubule networks to reach the NPC. During this process, CypA binds on the surface of the capsid to stabilize it and avoid targeting by cellular restriction factors. Subsequently, multiple copies of NUP358 filaments of the NPC capture the capsid from the cytosol, and facilitate docking of the virus core on the NPC. The capsid interactions with NTR or other cellular factors (or both)—such as TRN-1 and the complex of CPSF6 and TRN-SR2—assist the viral core in passing through the selective central barrier of the NPC. In the late stage of capsid nuclear transport, the nuclear basket-residing NUP153 enables the virus core to translocate to the nuclear side. In the meantime, NUP153 may stabilize the capsid lattice to avoid being disrupted when passing through the NPC. From the nuclear side of the NPC, the accumulation of CPSF6 binding to the capsid mediates the release of the capsid from the nuclear basket area into deeper regions of the nucleus, which is potentially assisted by other nuclear factors such as SUN1/2. While trafficking through the cytoplasm to the nucleus, HIV-1 actively performs the reverse transcription of its genome. The completion of RT inside the nucleus, likely at regions close to the integration site, triggers the uncoating of capsid to release the viral DNA transcript for integration into the host genome (Figure 4B).

From the cytoplasm to the nucleus, a finely balanced integrity of the capsid plays a vital role in the early phases of HIV-1 life cycle. Premature uncoating or an overly stable capsid may lead to the reduction or failure of infection. Nuclear entry of the virus core through the NPC is no longer considered inconceivable. However, many mechanistic questions remain unaddressed. What is the driving force for the nuclear entry of the capsid? How does the intact capsid penetrate the densely packed, selective barrier of the NPC? How does the capsid move inside the nucleus and in what manner does the completion of RT trigger uncoating? Many host factors are involved in these processes, and their detailed roles are yet to be fully understood. In addition, it remains to be clarified whether the virus genome has additional nuclear transport routes that are independent of the NPC-mediated pathway.

## Data Availability

Not applicable.
